# Risk-factor model for postpartum hemorrhage after cesarean delivery: a retrospective study based on 3498 patients

**DOI:** 10.1038/s41598-022-23636-5

**Published:** 2022-12-21

**Authors:** Jun Gong, Zhi Chen, Yi Zhang, Yi-yun Liu, Jun-cai Pu, Chun-yan Xiong, Si-wen Gui, Xiao-ling He, Hui-lai Wang, Xiao-gang Zhong

**Affiliations:** 1 Department of Information Center, The University-Town Hospital of Chongqing Medical University, Chongqing, 401331 China; 2 Medical Data Science Academy, Chongqing Medical University, Chongqing, 400016 China; 3 Department of Obstetrics and Gynecology, The First Affiliated Hospital of Chongqing Medical University, Chongqing, 400016 China; 4 School of Public Health and Management, Chongqing Medical University, Chongqing, 400016 China; 5 NHC Key Laboratory of Diagnosis and Treatment On Brain Functional Diseases, The First Affiliated Hospital of Chongqing Medical University, Chongqing, 400016 China; 6 Department of Obstetrics and Gynecology, The University-Town Hospital of Chongqing Medical University, Chongqing, 401331 China; 7 Key Laboratory of Psychoseomadsy, Stomatological Hospital of Chongqing Medical University, Chongqing, 401147 China; 8 College of Basic Medicine, Chongqing Medical University, Chongqing, 400016 China

**Keywords:** Diseases, Risk factors

## Abstract

This study aimed to investigate the risk factors of patients with postpartum hemorrhage (PPH) after cesarean delivery (CD) and to develop a risk-factor model for PPH after CD. Patients were selected from seven affiliated medical institutions of Chongqing Medical University from January 1st, 2015, to January 1st, 2020. Continuous and categorical variables were obtained from the hospital’s electronic medical record systems. Independent risk factors were identified by univariate analysis, least absolute shrinkage and selection operator and logistic regression. Furthermore, logistic, extreme gradient boosting, random forest, classification and regression trees, as well as an artificial neural network, were used to build the risk-factor model. A total of 701 PPH cases after CD and 2797 cases of CD without PPH met the inclusion criteria. Univariate analysis screened 28 differential indices. Multi-variable analysis screened 10 risk factors, including placenta previa, gestational age, prothrombin time, thrombin time, fibrinogen, anemia before delivery, placenta accreta, uterine atony, placental abruption and pregnancy with uterine fibroids. Areas under the curve by random forest for the training and test sets were 0.957 and 0.893, respectively. The F1 scores in the random forest training and test sets were 0.708. In conclusion, the risk factors for PPH after CD were identified, and a relatively stable risk-factor model was built.

## Introduction

Postpartum hemorrhage (PPH) is a severe complication during delivery. It is defined by The American College of Obstetricians and Gynecologists as the cumulative blood loss greater than or equal to 1000 ml or accompanied by symptoms or signs of hypovolemia within 24 h after birth (including intrapartum loss) regardless of route of delivery^1^, and is one of the main causes of global maternal mortality. According to the World Health Organization, PPH is associated with about 20% of maternal deaths annually^2^. In the United States, the maternal mortality rate attributable to PPH is 11.2%^3^. In China, the maternal mortality rate is 0.183/1000^4^, with PPH accounting for one-third of maternal deaths. In developing countries, the mortality rate of PPH is higher^3^, and the numbers are on the rise^5–7^.

Cesarean delivery (CD) is a predominant, independent risk factor for PPH^8^. In Israel, the rate of PPH after CD is 9.6%^9^, while 6.5% in China^10^. In the United States and India, blood transfusion rates after CD are 3.2% and 12.2%, respectively^11,12^. Therefore, the risk factors of PPH after CD should be explored for early identification and to develop a risk-factor model for PPH after CD. Extensive studies have evaluated the risk factors for PPH after CD^13–16^, and few of them have integrated these risk factors to build PPH risk-factor models. Furthermore, these studies have drawbacks, such as small sample sizes or low areas under the curve (AUC)^17–19^.

Machine learning (ML) is a new artificial intelligence discipline that is widely used to inform the management of diseases in the early stages^20–22^. For instance, Zheutlin^19^ established a PPH prediction model using a gradient boosted decision tree and achieved an AUC value of 0.71. Segar^21^ developed a novel machine learning-derived model to predict heart failure in diabetic patients and attained an AUC value of 0.74. Kang^18^ used a support vector machine and random forest models to screen septic shock in an emergency department with an AUC value of 0.83. Therefore, the aim of this study was to use ML algorithms to identify risk factors and build a risk-factor model for PPH after CD.

## Methods

### Study participants

Data were obtained from the medical big data platform of the medical data science academy, Chongqing Medical University (Chongqing, China), which includes seven medical institutions. All the seven medical institutions are affiliated hospitals or teaching hospitals of Chongqing Medical University and operate in a similar manner. The CD electronic medical data were collected from January 1st, 2015, to June 1st, 2020. Blood loss volume data were extracted from the electronic medical record. Briefly, blood loss volume was quantified in all cases by measuring the blood collected in the suction apparatus and by weighing lap pads, towels, gauzes and drapes. A coagulation examination is a routine assay after the patients are admitted to the hospitals. The inclusion criteria for this study were as follows: (1) Patients with CD; (2) Gestational age > 20 weeks; (3) Age ≥ 18 years. The exclusion criteria were as follows: (1) Patients with coagulation dysfunction^23–25^; (2) Patients with preoperative anticoagulant therapy and hemorrhagic diseases^24,25^; (3) Those missing all clinical information. This study protocol was reviewed and approved by the Ethics Committee of Chongqing Medical University, and with its approval, this study required no informed consent. All methods were performed in accordance with the Declaration of Helsinki and the relevant guidelines.

### Potential risk factors

During hospitalization, the clinical information of the patients, including laboratory examination records, imaging examination records, diagnosis and treatment process, were recorded on the medical big data platforms. Most of the variables were obtained before the CD, and their definition and access methods are shown in Supplementary Table 1. Based on the previous studies^8,18,26–30^, a total of 56 potential risk factors were obtained from the medical record systems. Of these, 10 factors had a missing rate of more than 30% and were excluded. Therefore, 46 factors were collected, including all admission first blood routine examination indices, coagulation indices and pregnancy indices. These included gravidity, number of previous deliveries, number of CD, prothrombin time (PT), thrombin time (TT), activated partial thromboplastin time (APTT), fibrinogen, neutrophil ratio (NEUT%), neutrophil count (NEUT#), monocyte ratio (MONO%), monocyte count (MONO#)), basophil ratio (BASO%), basophil count (BASO#), eosinophil ratio (EO%), eosinophil count (EO#), lymphocyte ratio (LYMPH%), lymphocyte count (LYMPH#), white blood cell count (WBC), red blood cell count (RBC), mean corpuscular volume (MCV), hemoglobin concentration (HGB), mean corpuscular hemoglobin (MCH), mean corpuscular hemoglobin concentration (MCHC), platelet count (PLT), mean platelet volume (MPV), platelet distribution width (PDW), platelet larger cell ratio (P-LCR), coefficient variation of red blood cell volume distribution width (RDW-CV), anemia before delivery, thrombocytopenia, gestational age, gestational hypertension, gestational diabetes mellitus, pregnancy with uterine fibroids, amniotic fluid index (AFI), estimated neonatal weight, preeclampsia, placental abruption, placenta previa, pre-labor rupture of membranes, uterine rupture, umbilical cord around the neck, placenta accreta, uterine atony, anesthesia, and pelvic adhesion.

### Statistical analysis

All statistical analyses were performed in R for windows (version 3.6.1, https://www.r-project.org/) and SPSS 24.0 (IBM Corporation, Armonk, NY, USA). Data were presented as counts with percentages for categorical variables, median with inter-quartile range (IQR) or mean with standard deviation for continuous variables. Variables with missing rate of more than 30% were excluded, while the miss-forest algorithm was used to fill the variables with missing rates of less than 30%. Propensity score matching (PSM) was used to balance the large difference in proportions between all CD with PPH patients and all CD without PPH patients at a ratio of 1 to 4; and maternal age and BMI were used as matching factors. Moreover, participants were randomized into the training and test sets using a random number table. The Mann–Whitney *U* test and *T*-test were used to analyze continuous variables, whereas the Chi-square test was used for all categorical variables. For multi-variable analysis, the least absolute shrinkage and selection operator (LASSO) and logistic regression analysis were performed. Variance inflation factor (VIF) was used to assess multi-collinearity between variables, with VIF > 10 indicating collinearity. Then, extreme gradient boosting (XGBoost), random forest (RF), classification and regression trees (CART) and artificial neural network (ANN) were used to develop a risk-factor model. The model’s performance was assessed by its specificity, precision, recall, F1 score and AUC; a larger value indicates a higher performance^31^. Significance was established at *P* < 0.05. Evaluation metrics for model performance were as follows:

$$ Precision = \frac{TP}{{TP + FP}} $$

$$ Recall = \frac{TP}{{TP + FN}} $$

$$ F1 = \frac{2 \times Precision \times Recall}{{Precision + Recall}} $$

TP: true positive numbers; TN: true negative numbers; FP: false positive numbers; FN: false negative numbers.

## Results

**Figure 1:**
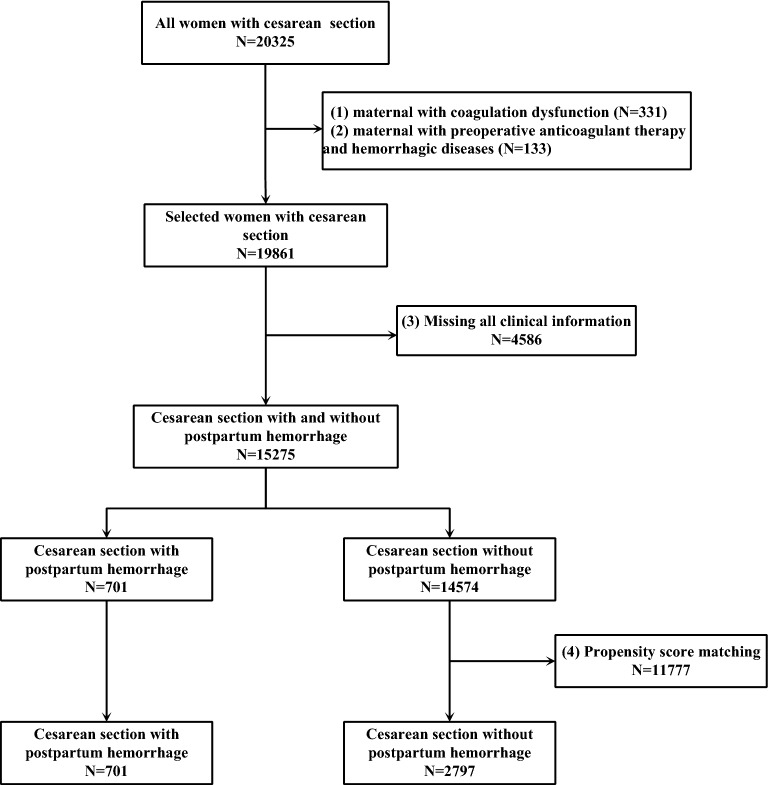
Flowchart for screening study participants.

A total of 15,275 patients, including 701 patients with PPH after CD and 14,574 patients without PPH after CD, met the inclusion criteria. The CD rate in these hospital units was 33.36%. Propensity score matching (PSM) was used to match 2797 patients without PPH after CD (control group) with the 701 patients with PPH after CD (study group). Eventually, a total of 3498 participants, including 701 (20%) patients with PPH after CD and 2797 (80%) patients without PPH after CD, were included in this study. There were 3457 (98.83%) patients of the Chinese Han nationality. The other nationalities were represented by 41 (1.17%) patients, with nine patients in the study group and 32 in the control group. The flow chart for screening the study participants is shown in Fig. 1.

The patients were randomized into the training set (n = 2448, 70%) and the test set (n = 1050, 30%). The training set had 477 patients with PPH after CD and 1971 patients without PPH after CD. The test set had 224 patients with PPH after CD and 826 patients without PPH after CD. Univariate analysis revealed 28 significant variables between the study and the control groups, while 18 variables were insignificant (Table 1). Statistically different variables were further identified by LASSO to find the optimal value of lambda by balancing accuracy and simplicity. The log of the optimal value of lambda was 10 (Supplementary Fig. 1). Thus, 10 significant variables (Table 2) were retained and analyzed by logistic analysis. The risk factors associated with PPH after CD included pregnancy with uterine fibroids, anemia before delivery, placenta previa, placenta accreta, placental abruption, uterine atony, small for gestational age, prolonged PT, prolonged TT, and low fibrinogen.

**Table 1:** Univariate analysis of CD-PPH and CD no-PPH in the training set.

Variables	CD-PPH (n = 477)		CD-no PPH(n = 1971)		*χ*^*2*^*/Z*	*P* value
	Missing rate(%)	N (%) or Quartile	Missing rate(%)	N (%) or Quartile		
**Demographic characteristics**						
Age	0.0	30.00 (27.00, 34.00)	0.0	30.00 (27.00, 34.00)	− 0.742	0.458
BMI	1.6	26.96 (26.16, 27.86)	2.3	26.84 (25.56, 28.35)	** − **1.213	0.225
**History**						
Gravidity	0.0	2.00 (1.00,4.00)	0.0	2.00 (1.00,3.00)	36.824	< 0.001
Number of previous deliveries	0.0	0.0 0 (0.00,1.00)	0.0	0.00 (0.00,1.00)	66.473	< 0.001
Number of CD	0.0	0.0 0 (0.00,1.00)	0.0	0.00 (0.00,1.00)	33.856	< 0.001
**Coagulation examination**						
PT (s)	5.2	11.50 (10.80,12.10)	10.1	10.95 (10.50,11.60)	** − **10.484	< 0.001
TT (s)	6.3	16.20 (15.20,17.20)	10.9	15.50 (13.50,16.60)	** − **8.321	< 0.001
APTT (s)	4.8	29.20 (26.20,31.60)	9.7	27.50 (25.60,30.20)	** − **5.970	< 0.001
Fibrinogen (g/L)	5.7	4.06 (3.51,4.54)	9.2	4.20 (3.68,4.72)	** − **4.506	< 0.001
**Blood routine examination**						
NEUT% (%)	9.6	76.12 (71.70,79.20)	11.3	75.30 (71.00,79.10)	** − **2.172	0.030
NEUT# (10^9/L)	18.7	6.57 (5.28,7.80)	19.0	6.28 (5.00,7.79)	** − **2.416	0.016
MONO% (%)	7.1	5.80 (4.50,6.80)	18.0	5.6 (4.5,6.8)	** − **0.534	0.593
MONO# (10^9/L)	9.2	0.49 (0.36,0.59)	18.7	0.47 (0.35,0.61)	** − **1.075	0.282
BASO% (%)	8.0	0.20 (0.10,0.30)	15.6	0.20 (0.10,0.30)	** − **1.575	0.115
BASO# (10^9/L)	9.4	0.02 (0.01,0.02)	17.3	0.02 (0.01,0.02)	** − **0.240	0.811
EO% (%)	8.8	0.70 (0.40,1.00)	15.2	0.60 (0.40,1.10)	** − **0.737	0.461
EO# (10^9/L)	9.4	0.06 (0.03,0.08)	16.5	0.05 (0.03,0.09)	** − **0.334	0.738
LYMPH# (10^9/L)	9.0	1.43 (1.19,1.72)	18.8	1.46 (1.21,1.77)	** − **1.797	0.072
LYMPH% (%)	7.5	17.31 (14.00,21.00)	17.8	18.00 (14.42,21.85)	** − **2.577	0.010
WBC (10^9/L)	9.4	8.79 (7.08,9.97)	17.0	8.37 (6.94,10.09)	** − **1.837	0.066
RBC (10^12/L)	9.0	3.84 (3.55,4.06)	20.0	3.94 (3.68,4.23)	** − **5.845	< 0.001
MCV (fl)	8.6	92.30 (88.00,95.20)	16.9	91.50 (87.20,95.00)	** − **1.992	0.046
HGB (g/L)	5.5	116.00 (105.00,124.00)	6.5	118.00 (109.00,128.00)	** − **4.820	< 0.001
MCH (pg)	8.8	30.60 (28.80,31.59)	16.1	30.30 (28.55,31.70)	** − **0.694	0.488
MCHC (g/L)	10.1	331.29 (324.00,336.40)	6.2	330.00 (322.00,338.00)	** − **0.853	0.394
PLT (10^9/L)	7.3	178.00 (143.74,208.00)	6.6	182.00 (148.00,220.00)	** − **2.172	0.030
MPV (fl)	10.5	10.99 (10.40,11.90)	6.4	11.00 (10.00,12.10)	** − **0.203	0.839
PDW (%)	8.0	16.00 (14.29,16.87)	5.4	16.40 (15.20,17.00)	** − **3.893	< 0.001
P-LCR (%)	20.3	33.50 (30.16,39.45)	16.7	34.50 (27.82,41.68)	** − **0.282	0.778
RDW-CV (%)	8.4	14.21 (13.70,15.27)	9.5	14.10 (13.40,15.00)	** − **3.610	< 0.001
**Indices during the pregnancy**						
Gestational age (weeks)	10.3	37.00 (36.00,38.00)	20.0	38.57 (38.00,39.00)	** − **14.761	< 0.001
Anemia before delivery	0.0	167 (35.01)	0.0	239 (12.13)	145.386	< 0.001
Thrombocytopenia	0.0	14 (2.94)	0.0	36 (1.83)	2.359	0.147
Gestational hypertension	0.0	20 (4.19)	0.0	48 (2.44)	4.393	0.043
Gestational diabetes mellitus	0.0	109 (22.85)	0.0	374 (18.98)	3.643	0.056
Pregnancy with uterine fibroids	0.0	17 (3.56)	0.0	10 (0.51)	32.895	< 0.001
AFI (mm)	24.3	125.49 (110.34,135.32)	22.7	118.36 (103.59,132.39)	** − **4.958	< 0.001
Estimated neonatal weight (g)	7.5	3200 (2972.40,3400.00)	14.6	3300.00 (3004.05,3600.00)	** − **5.960	< 0.001
Preeclampsia	0.0	23 (4.82)	0.0	62 (3.15)	3.219	0.093
Placental abruption	0.0	18 (3.77)	0.0	4 (0.20)	54.979	< 0.001
Placenta previa	0.0	171 (35.38)	0.0	55 (2.79)	500.879	< 0.001
Pre-labor rupture of membranes	0.0	45 (9.43)	0.0	2 (0.10)	177.632	< 0.001
Uterine rupture	0.0	11 (3.31)	0.0	48 (2.44)	0.027	1.000
Umbilical cord around the neck	0.0	63 (13.21)	0.0	250 (12.68)	0.094	0.760
Placenta accreta	0.0	221 (46.33)	0.0	110 (5.58)	545.418	< 0.001
Uterine atony	0.0	25 (5.24)	0.0	12 (0.61)	55.361	< 0.001
Anesthesia (general)	0.0	36 (7.55)	0.0	99 (5.02)	4.697	0.034
Pelvic adhesion	0.0	33 (6.92)	0.0	157(7.97)	0.599	0.505

N, number of samples; %, percent; Quartile, inter-quartile range between 25th and 75th percentiles; *χ*^*2*^, value for chi-square test; Z, value for Mann–Whitney U test.

**Table 2:** Factors associated with CD-PPH in the training set.

Variables	B value	P value	Adjusted OR	95% CI	VIF
Pregnancy with uterine fibroids	1.709	0.001	5.524	1.987,15.362	1.028
Anemia before delivery	1.090	< 0.001	2.974	2.169,4.079	1.059
Placenta previa	2.219	< 0.001	9.200	6.174,13.708	1.180
Placenta accreta	2.554	< 0.001	12.852	9.321,17.721	1.109
Placental abruption	2.633	< 0.001	13.918	3.287,58.936	1.052
Uterine atony	2.318	< 0.001	10.153	4.291,24.022	1.021
Gestational age	-0.236	< 0.001	0.790	0.739,0.844	1.076
PT	0.488	< 0.001	1.628	1.399,1.896	1.193
TT	0.146	< 0.001	1.157	1.083,1.237	1.142
Fibrinogen	-0.339	< 0.001	0.712	0.607,0.836	1.057

OR, odds ratio; 95% CI, 95% confidence interval; VIF, variance inflation factor.

**Figure 2:**
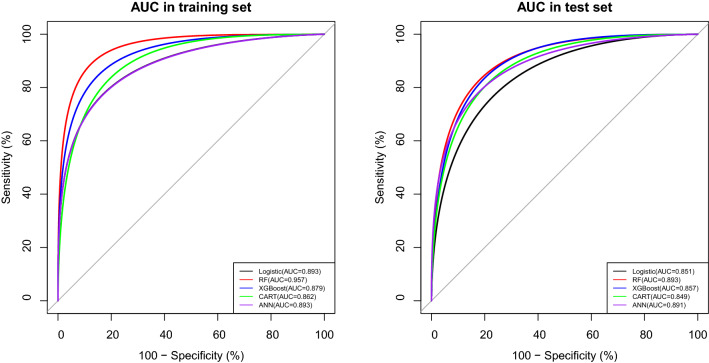
AUC values for five models in the training and test sets.

To establish a risk-factor model, the 10 independent risk factors were used as input variables in the ML algorithm, with PPH after CD as the outcome event (yes = 1, no = 0). In the training set, Logistic, XGBoost, RF, CART, and ANN models had AUC of 0.893 (0.875–0.911), 0.879 (0.859–0.898), 0.957 (0.950–0.965), 0.862 (0.842–0.883), and 0.893 (0.875–0.911), respectively, and 0.851 (0.823–0.880), 0.857 (0.828–0.887), 0.893 (0.867–0.918), 0.849 (0.818–0.880), and 0.891 (0.866–0.916), respectively, in the test set (Table 3, Fig. 2). The F1 score of the RF model in the training and test sets was 0.708 (Table 3). Since multiple variables were present during CD, we also constructed the risk-factor model based on the potential risk factors before CD. The results were similar to those obtained by the RF model (Supplementary Table 2).

**Table 3:** Evaluation of model fitness in training and test sets.

Data	Model	Precision	Recall	F1 score	AUC
Training set	Logistic	0.613	0.780	0.686	0.893 (0.875–0.911)
	XGBoost	0.716	0.728	0.722	0.879 (0.859–0.898)
	RF	0.570	0.933	0.708	0.957 (0.950–0.965)
	CART	0.551	0.797	0.651	0.862 (0.842–0.883)
	ANN	0.598	0.793	0.682	0.893 (0.875–0.911)
Test set	Logistic	0.571	0.714	0.635	0.851 (0.823–0.880)
	XGBoost	0.655	0.719	0.685	0.857 (0.828–0.887)
	RF	0.664	0.759	0.708	0.893 (0.867–0.918)
	CART	0.540	0.790	0.641	0.849 (0.818–0.880)
	ANN	0.578	0.813	0.675	0.891 (0.866–0.916)

**Figure 3:**
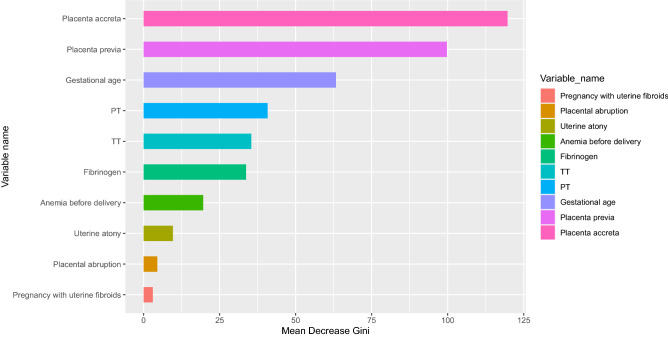
Variable importance score in the RF model.

Evaluation indices indicated that RF outperformed the other models. Based on the gini coefficient, the 10 variables were ranked as follows: placenta accreta, placenta previa, gestational age, PT, TT, fibrinogen, anemia before delivery, uterine atony, placental abruption and pregnancy with uterine fibroids, (Fig. 3). The web-based tool was developed based on these 10 risk factors (https://cqmugj.shinyapps.io/pph_after_cd/). Furthermore, another web-based tool was also constructed based on seven risk factors before CD (https://cqmugj.shinyapps.io/pph_after_cd_2/).

## Discussion

In this study, we report a risk-factor model for PPH after CD based on the ML algorithm. 46 variables were collected and analyzed by univariate analysis. Among them, 28 variables showed a significant difference between the two groups, and the 10 independent risk factors were filtered in a multi-variable analysis. Finally, five risk-factor models were successfully developed to discriminate between PPH after CD and no-PPH after CD.

Many studies have investigated PPH after CD^13–16^. In America, a study revealed that placenta previa is a risk factor for PPH after CD^15^. According to an Australian study, general anesthesia is closely correlated with PPH^16^. Furthermore, many studies found that pregnant women with hypertensive disorders, and placenta accreta spectrum disorders were more likely to suffer from PPH^10,13–16^. However, a limited number of studies have integrated these factors to develop a risk-factor model^19^.

In this study, placenta previa, gestational age, PT, TT, fibrinogen, anemia before delivery, placenta accreta, uterine atony, placental abruption, and pregnancy with uterine fibroids were identified as risk factors for PPH after CD. Most of theses independent risk factors have previously been reported^8,10,13,14,17,29^. For example, both the study from the Southern Ethiopia and Mali showed that antepartum anemia mothers are more likely to suffer from PPH^32,33^. The effect of placenta previa has also been reported in many studies^15,34,35^. A study from China found the patients with complete placenta previa had higher risk of PPH than those incomplete or no placenta previa^34^. Therefore, the guidelines^36^ usually recommend planned cesarean delivery for better maternal outcomes^35^. Placenta accreta refers to the penetration of placental villi into part of the muscular layer of the uterine wall. The implanted part of the placenta fails to detach itself during childbirth. Manual separation of the placenta may damage the myometrium, resulting in severe bleeding, perforation or maternal death^37^. Placental abruption is characterized by the partial or total detachment of the placenta from the uterine wall before the delivery of the fetus. In China, placental abruption has an incidence of 0.46%–2.1%^38^ and 0.3%–1.2% in other countries (US, Canada, Sweden, Norway, Denmark, Finland and Spain)^39^. It is a critical complication during the pregnancy (after 20 weeks) or delivery^40^. Uterine atony is one of the most common causes of PPH. With prolonged labor, some pregnant women lose effective pressure on blood vessels, resulting in PPH^41^. Previous studies have shown small for gestational age is associated with PPH^42,43^. Furthermore, premature delivery with gestational hypertension, severe preeclampsia, and placenta previa can significantly increase the risk of PPH^44–46^. Fibrinogen is involved in platelet aggregation during secondary homeostasis. High prenatal fibrinogen levels are associated with low PPH incidences^47,48^. However, the effects of uterine fibroids on the risk of PPH after CD remain poorly understood. Some studies reported that uterine fibroids do not increase the risk of obstetric complications^49,50^. In contrast, other studies found that uterine fibroids with a diameter of over 5 cm are a risk factor for PPH after CD^30,51^. Our results were in accordance with the latter findings. A possible explanation is that uterine fibroids prevent uterine contractility, and those with a larger diameter and specific position affect uterine contractions, increase dystocia, and increase PPH risk^52^. The activities of coagulation factors I, II, V, VII and X in plasma can be reflected by PT and TT. We found that PT and TT were significantly prolonged in the PPH after CD group, indicating impaired coagulation in the study group compared to the control group, which may be associated with massive hemorrhage^53^.

Studies have developed risk-factor models for PPH after CD. Zheutlin’s model^19^, which included 24 unique features from the United States, had an AUC of 0.71 (95%CI: 0.69–0.72), while Wu’s^54^ model, which was built using 35 radiomic features, had an AUC value of 0.83 (95% CI: 0.75–0.91). Several studies have also constructed prediction models for blood transfusion after CD. Ahmadzia’s^8^ model, which was based on prenatal and intrapartum variables from 19 medical institutions in the United States, demonstrated an AUC value of 0.83 (95% CI: 0.81–0.84). Kang’s^18^ model, which involved 5 risk factors from South Korea, had an AUC value of 0.83 (95% CI: 0.70–0.92). The parameters of these models are shown in Supplementary Table 3. Compared to the aforementioned models, the risk-factor model constructed using RF in this study performed better.

Based on 10 influencing factors, we developed two web-based tools for PPH after CD, which could be applied in the participating hospital units. Healthcare workers or patients will get a probability score of PPH by filling all or some of the 10 factors. For high-risk patients, reasonable measures could be taken for PPH prevention, such as ameliorating of patient’s anemia, correcting patient’s coagulation disorders with medication before CD, and preparing additional plasma or serum in advance. Many of the 10 influencing factors could not be modified. Therefore, further studies should focus on improving the performance of this model by increasing the sample size, supplementing other types of patient populations (such as patients with coagulation disorders), and screening other influencing factors (such as age and ethnicity).

In conclusion, we identified several risk factors for PPH after CD, including pregnancy with uterine fibroids, anemia before delivery, placenta previa, placenta accreta, placental abruption, uterine atony, small for gestational age, prolonged PT, prolonged TT, and low fibrinogen. Furthermore, we developed a risk-factor model to predict the risk of PPH after CD.

## Limitations

This study has several limitations. First, all of the data is obtained from hospital units, in southwest of China, which may have caused a selection bias and a clustering effect in our model. Second, variables with a missing rate ≥ 30% were not included in this study. Therefore, further analysis should be performed to establish whether the excluded factors are associated with PPH after CD. Third, some variables, such as gestational age, had a relatively high missing rate. Although we used the miss-forest algorithm to fill in the missing data, it still lower the validity of this model. Fourth, some risk factors, such as PPH history, urgent or elective CDs, cervical dilation, and first or second stages of delivery, the fibroids sizes, could not be obtained from the platform, reducing the clinical utility of the model. Fifth, since this study did not strictly adhere to the TRIPOD statement, there was no external independent data to validate our results, which limits the generalization ability of the model. Sixth, most of the variables in the final model could not be modified, and just a few variables were available during the CD, thus, it is difficult to apply the model in clinical decision-making. Admittedly, the protocols were not the same in all seven hospitals, and we did not distinguish between elective and urgent surgeries, induction versus spontaneous onset according to labour stage. Eventually, this study lacked data regarding indications for CD. Therefore, there is heterogeneity in our study and more studies are needed to support our results.

## Supplementary Information

Supplementary Information.

## Data Availability

The datasets used and/or analyzed during the current study available from the corresponding author on reasonable request.
