# Supplementary material for: Risk-factor model for postpartum hemorrhage after cesarean delivery: a retrospective study based on 3498 patients
Source: Sci Rep. 2022 Dec 21;12:22100. doi: 10.1038/s41598-022-23636-5 (PMC9772352; doi:10.1038/s41598-022-23636-5)

Supplementary Materials

A risk-factors model for postpartum hemorrhage after cesarean delivery: a retrospective study based on 3498 patients

Jun Gong[1,2], Zhi Chen[3], Yi Zhang[4], Yi-yun Liu[5], Jun-cai Pu[5], Chun-yan Xiong[6], Si-wen Gui[5], Xiao-ling He[3], Hui-lai Wang[1,2*] & Xiao-gang Zhong[2,7,8*]

[1]Department of Information Center, The University-Town Hospital of Chongqing Medical University, Chongqing, 401331, China.

[2]Medical Data Science Academy, Chongqing Medical University, Chongqing, 400016, China.

[3]Department of Obstetrics and Gynecology, The First Affiliated Hospital of Chongqing Medical University, Chongqing, 400016, China.

[4]School of Public Health and Management, Chongqing Medical University, Chongqing, 400016, China.

[5]NHC Key Laboratory of Diagnosis and Treatment on Brain Functional Diseases, The First Affiliated Hospital of Chongqing Medical University, Chongqing, 400016, China.

[6]Department of Obstetrics and Gynecology, The University-Town Hospital of Chongqing Medical University, Chongqing, 401331, China.

[7]Key Laboratory of Psychoseomadsy, Stomatological Hospital of Chongqing Medical University, Chongqing, 401147, China.

[8]College of Basic Medicine, Chongqing Medical University, Chongqing 400016, China.

Supplementary Table 1. The definition and access methods of variables.

Items	Variables	Definition	Access methods
**1**	Gravidity	The number of pregnancies, current and past, regardless of the pregnancy outcome.	History, electronic medical record
**2**	Number of previous deliveries	The number of previous deliveries a woman has experienced.	History, electronic medical record
**3**	Number of cesarean deliveries	The number of previous cesarean deliveries a woman has experienced.	History, electronic medical record
**4**	Prothrombin time	The addition of thrombin and calcium ions to plasma converts prothrombin to thrombin, resulting in the time required for plasma coagulation.	Coagulation examination
**5**	Thrombin time	The time of blood clotting after the addition of standardized thrombin to plasma.	Coagulation examination
**6**	Activated partial thromboplastin time	Aptt reagent was added to the plasma, and factor XII was activated by clay. Finally, calcium ion was added to observe the time required for plasma coagulation.	Coagulation examination
**7**	Fibrinogen	Fibrinogen is a glycoprotein ($\alpha 2 \beta 2 \gamma 2$) synthesized and secreted by liver cells. It is an important protein fibrin involved in coagulation and hemostasis.	Coagulation examination
**8**	Neutrophil ratio	The percentage of neutrophils in white blood cells.	Blood routine examination
**9**	Neutrophil count	The number of neutrophils per unit volume of blood.	Blood routine examination
**10**	Monocyte ratio	The percentage of monocytes in white blood cells.	Blood routine examination
**11**	Monocyte count	The number of monocytes per unit volume of blood.	Blood routine examination
**12**	Basophil ratio	The percentage of basophils in white blood cells.	Blood routine examination
**13**	Basophil count	The number of basophils per unit volume of blood.	Blood routine examination
**14**	Eosinophil ratio	The percentage of eosinophils in white blood cells.	Blood routine examination
**15**	Eosinophil count	The number of eosinophils per unit volume of blood.	Blood routine examination
**16**	Lymphocyte ratio	The percentage of lymphocytes in white blood cells.	Blood routine examination
**17**	Lymphocyte count	The number of lymphocytes per unit volume of blood.	Blood routine examination

**18**	White blood cell count	The number of white blood cells per unit volume of blood.	Blood routine examination
**19**	Red blood cell count	The number of red blood cells per unit volume of blood.	Blood routine examination
**20**	Mean corpuscular volume	The mean volume of the individual red blood cells in the human body.	Blood routine examination
**21**	Hemoglobin concentration	The amount (grams) of hemoglobin per unit volume of blood.	Blood routine examination
**22**	Mean corpuscular hemoglobin	The average amount of hemoglobin per red blood cell in peripheral blood.	Blood routine examination
**23**	Mean corpuscular hemoglobin concentration	The amount (grams) of hemoglobin per liter of red blood cells, after removing plasma, white blood cells, and platelets.	Blood routine examination
**24**	Platelet count	The number of platelets per unit volume of blood.	Blood routine examination
**25**	Mean platelet volume	The mean volume of individual platelets in peripheral blood.	Blood routine examination
**26**	Platelet distribution width	Coefficient of variation in measured platelet volume size.	Blood routine examination
**27**	Platelet larger cell ratio	The percentage of large platelets in the blood.	Blood routine examination
**28**	Coefficient variation of red blood cell volume distribution width	Coefficient of variation in red blood cell volume.	Blood routine examination
**29**	Anemia before delivery	The last blood routine examination (before giving birth) results showed the hemoglobin concentration in peripheral blood of pregnant women is less than 110g/L.	Blood routine examination, electronic medical record
**30**	Thrombocytopenia	The number of platelets per unit volume of blood is less than 100×10^9/L.	Blood routine examination, electronic medical record
**31**	Gestational age (weeks)	Gestational age (written with both weeks and days, e.g., 39 weeks and 0 days) is calculated using the obstetrical EDD based on the following formula: Gestational Age = (280 - (EDD - Reference Date))/ 7.	History of present illness, ultrasound
**32**	Gestational hypertension	Hypertension with systolic blood pressure ≥140 mm Hg and/or diastolic blood pressure ≥90mmHg developed after 20 weeks of gestation and returned to normal within 12 weeks after delivery; negative urine protein.	History, vital signs, urine routine examination, electronic medical record
**33**	Gestational diabetes mellitus	A 75 grams oral glucose tolerance test (OGTT) that resulted in fasting glucose	75 grams oral glucose tolerance

		⩾ 5.1 mmol/l, 1-h ⩾ 10 mmol/l, or 2-h ⩾ 8.5 mmol/l.	test, electronic medical record
**34**	Pregnancy with uterine fibroids	Concomitant uterine fibroids were identified in pregnant women.	Ultrasound, electronic medical record
**35**	Amniotic fluid index	During ultrasonic examination, the uterus was divided into four quadrants by the umbilical line and albino, and the vertical diameter line of the largest amniotic pool in each quadrant was measured. The sum of the four was the amniotic fluid index.	Ultrasound
**36**	Estimated neonatal weight	Neonatal weight was estimated based on the indices of pregnant women's uterine height and abdominal circumference.	Tape measurement
**37**	Preeclampsia	After 20 weeks of gestation, systolic blood pressure ≥140mmHg and/or diastolic blood pressure ≥90mmHg, accompanied by urinary protein ≥0.3g/24h, or random positive urinary protein, or combined with abnormal laboratory indicators of liver, kidney, blood system, or central nervous system abnormalities or visual impairment.	History, vital signs, urine routine examination, blood routine examination, urine protein, liver function tests, kidney function tests, fundus examination, electronic medical record
**38**	Placental abruption	Placental separation from the uterus with bleeding (concealed or vaginal) before fetal birth, with or without maternal/fetal compromise.	History, vital signs, laboratory examinations, ultrasound combined with the visual observation during operation, electronic medical record
**39**	Placenta previa	After 28 weeks of gestation, the placenta is lower than the fetal exposure, attached to the lower segment of the uterus, lower margin to reach or cover the cervical opening.	Ultrasound, electronic medical record
**40**	Pre-labor rupture of membranes	Spontaneous rupture of membranes that occurs before the onset of labor.	Vital signs, physical examination, ultrasound, examination sample of vaginal

			discharge, electronic medical record
**41**	Uterine rupture	Rupture of uterine body or lower uterine segment during late pregnancy or delivery.	History, vital signs, ultrasound, combined with clinical features of operation, electronic medical record
**42**	Umbilical cord around the neck	The umbilical cord was wrapped around the fetus's neck.	Visual observation during operation, electronic medical record
**43**	Placenta accreta	Penetration of the placenta into the musculum of the uterus, even penetrate through the musculum to reach the serous layer, or penetrates through the serous layer to erode the intestine or bladder; or simply the adhesion of the texture of the placenta to the uterine wall.	Clinical manifestation, ultrasound, MRI, electronic medical record
**44**	Uterine atony	Reduced intensity or frequency of uterine contractions.	Clinical manifestation, electronic medical record
**45**	Anesthesia	A reversible inhibition of function of the central and/or peripheral nervous system by drug or other means, characterized primarily by loss of sensation, especially pain.<br># It stands for (general anesthesia)/(general anesthesia+regional anesthesia) in our main text.	Clinical operation, clinical manifestation, electronic medical record
**46**	Pelvic adhesion	Inflammation of the tissues or organs in the pelvic cavity occurs after the infection, resulting in tissue congestion, edema, and increased secretions, which may produce adhesions to the uterus, fallopian tubes, ovaries, and fallopian tubes, collectively referred to as pelvic adhesion.	History, physical examination, laparoscopic investigation, hysterosalpingography, electronic medical record

Supplementary Table 2. Evaluation of the model excluding the indicators during CD

**Data**	**Model**	***Precision***	***Recall***	***F1***	***AUC***
Training set	Logistic	0.517	0.755	0.614	0.862 (0.842-0.882)
	XGBoost	0.514	0.767	0.616	0.854 (0.833-0.875)
	RF	0.509	0.964	0.667	0.951 (0.943-0.959)
	CART	0.485	0.782	0.599	0.860 (0.841-0.880)
	ANN	0.551	0.721	0.625	0.863 (0.843-0.883)
Test set	Logistic	0.571	0.714	0.635	0.851 (0.823-0.880)
	XGBoost	0.518	0.710	0.599	0.805 (0.768-0.841)
	RF	0.595	0.674	0.632	0.856 (0.827-0.885)
	CART	0.560	0.688	0.617	0.836 (0.806-0.866)
	ANN	0.517	0.750	0.612	0.852 (0.824-0.880)

Using 7 factors, including Pregnancy with uterine fibroids, Anemia before delivery, Placenta accreta, Gestational age, PT, TT, Fibrinogen

Supplementary Table 3. Parameters of previous studies

**Author**	**PMID**	**Model**	**Variables**	**AUC**
Zheutlin, A.B., et al	34405866	gradient boosting decision tree	24 unique features, including 7 were lab results, 6 were diagnoses, 4 were vital signs, 4 were demographic variables, and 3 were medications.	0.71 (95%CI, 0.69-0.72)
Wu, Q. et al	31767539	support vector machine	35 radiomic features	0.83 (95%CI, 0.75-0.91)
Ahmadzia, H. K et al	30551126	Multivariable logistic regression (antepartum model)	Maternal age, BMI at delivery, 3+ previous term deliveries, Gestational age <37 weeks, African American race, Insurance: govt vs. private, Insurance: none vs. private, Platelet count <150, Hematocrit <32, No previous CD, 2 previous CD,3+ previous CD, Hx heart disease, Gest. HTN/Preeclampsia, HELLP, Abruption antepart	0.77 (95% CI 0.75–0.78)
		Multivariable logistic regression (antepartum and intrapartum model)	Maternal age, BMI at delivery, 3+ previous term deliveries, Insurance: govt vs. private, Insurance: none vs. private, Platelet count <150, Hematocrit <32, No previous CD, 2 previous CD, Hx heart disease, Gest. HTN/Preeclampsia, Non-elective repeat CD, General anesthes, Abruption at time of delivery, Multiple gestation, Failure to progress, Eclampsia/HELLP, Placenta accreta, increta, or percre, Antibiotic use	0.83 (95% CI 0.81–0.84)
Kang J. et al	31997624	Multivariable logistic regression	Age, Previa, lacunae ,Hypoechoic layer, antierior placeta	0.83 (95% CI 0.70–0.92)

Supplementary Figure 1. The log of the optimal value of lambda by LASSO.

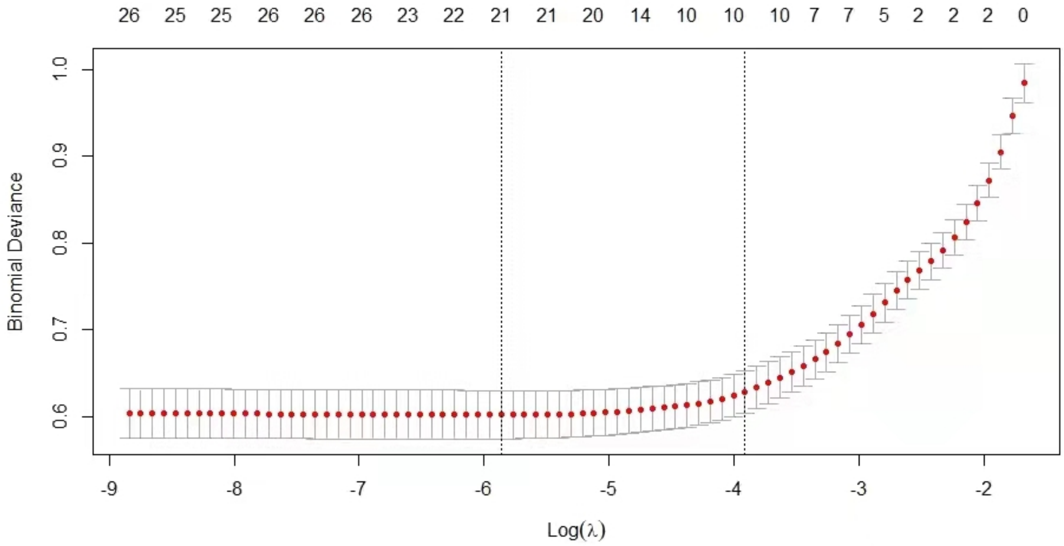

Supplement: Supplementary file 1 — Supplementary Information. [file 41598_2022_23636_MOESM1_ESM.pdf]
